# Characterization of RNA modifications in gastric cancer to identify prognosis‐relevant gene signatures

**DOI:** 10.1002/cam4.4861

**Published:** 2022-05-30

**Authors:** Qingchuan Chen, Zhouyang Liu, Yuen Tan, Siwei Pan, Wen An, Huimian Xu

**Affiliations:** ^1^ Department of Surgical Oncology The First Affiliated Hospital of China Medical University Shenyang China; ^2^ Department of Neurology The First Hospital of China Medical University Shenyang China

**Keywords:** alternative cleavage and polyadenylation, gastric cancer, prognosis, RNA modification, scoring system

## Abstract

**Background:**

Most human genes have diverse transcript isoforms, which mainly arise from alternative cleavage and polyadenylation (APA) at 3′ ends. N7‐methylguanosine (m^7^G) is also an essential epigenetic modification at the 5′ end. However, the contribution of these two RNA modifications to the development, prognosis, regulation mechanisms, and drug sensitivity of gastric cancer (GC) is unclear.

**Methods:**

The expression data of 2412 patients were extracted from 12 cohorts and the RNA modification patterns of 20 marker genes were systematically identified into phenotypic clusters using the unsupervised clustering approach. Following that, we developed an RNA modification model (RMscore) to quantify each GC patient's RNA modification index. Finally, we examined the correlation between RMscore and clinical features such as survival outcomes, molecular subtypes identified by the Asian Cancer Research Group (ACRG), posttranscriptional regulation, and chemotherapeutic sensitivity in GC.

**Results:**

The samples were categorized into two groups on the basis of their RMscore: high and low. The group with a low RMscore had a bad prognosis. Moreover, the low RMscore was associated with KRAS, Hedgehog, EMT, and TGF‐β signaling, whereas a high RMscore was related to abnormal cell cycle signaling pathway activation. The findings also revealed that the RMscore contributes to the regulation of the miRNA‐mRNA network. Drug sensitivity analysis revealed that RMscore is associated with the response to some anticancer drugs.

**Conclusions:**

The RMscore model has the potential to be a useful tool for prognosis prediction in patients with GC. A comprehensive investigation of APA‐RNA and m^7^G‐RNA modifications may reveal novel insights into the epigenetics of GC and aid in the development of more effective treatment strategies.

## Graphical abstract

We constructed an RNA modification model (RMscore) to quantify the RNA modification index of each GC patient. The RMscore model is a potentially useful tool to predict the prognosis of GC. Comprehensive analysis of APA‐RNA and m7G‐RNA modifications may provide novel insights into the epigenetics of GC and improve treatment strategies.

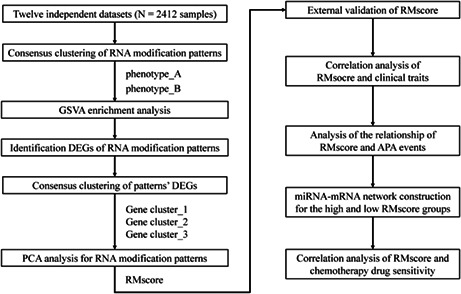

## 1 BACKGROUND

Gastric cancer (GC) is a significant contributor to the global burden of cancer disease, especially in Eastern Asian and Eastern European countries, ranking fifth for incidence and fourth for mortality among all cancers.^1^ The genesis and development of GC are reported to be because of the accumulation of epigenetic changes and gene mutations, which in turn lead to transcriptional or translational dysregulation.^2^, ^3^ In addition to genetic mutations, epigenetic modifications have been considered as another primary cause of tumorigenesis. They contribute significantly to the pathogenesis of cancer by inducing many unique biological behaviors of tumor cells.^4^ To investigate the unique expression patterns of genes associated with GC at the posttranscriptional levels, we observed the prognosis of GC patients from the perspective of alternative cleavage and polyadenylation (APA)‐RNA and N7‐methylguanosine (m^7^G)‐RNA modification.

The 5′ cap and 3′ poly(A) tail are two major common posttranscriptional modifications of mRNA, which are responsible for most of the various isoforms by generating alternative modification sites at the 5′ and 3′ ends.^5^ APA modification generates distinct 3′ ends during the maturation of pre‐mRNAs, and m^7^G is an essential modification against degradation at the 5′ ends. Furthermore, these two RNA modification mechanisms regulate mRNA splicing, cellular location, transcription, and translation in the mRNA life cycle.^6^, ^7^

Human genes (at least 70%) express multiple polyadenylation sites, and APA events are reported to be important triggers for many cancers.^8^, ^9^ Currently, four protein complexes and multiple protein monomers are known to participate in the modifications at the 3′ end. The protein complexes are composed of CPSF, CSTF, CFIm, and CFIIm, whereas the monomers include PABPs, PP1s, PAPOLG, RBBP6, and SYMPK.^6^, ^10^, ^11^ m^7^G is co‐transcribed at the 5′ cap during transcriptional initiation to ensure transcriptional stability and efficiency.^12^ A recently published study discovered the presence of m^7^G in mRNA and identified METTL1/WDR4 as an m^7^G writer complex, which affects the translation of mRNA.^7^ We selected 20 representative marker genes that were closely related to APA‐RNA and m^7^G‐RNA modifications.

Despite substantial studies on RNA modification, clinical evidence on the correlation between RNA modification and the development of cancer is insufficient. The analysis of several transcript isoforms produced from patterns of APA‐RNA and m^7^G‐RNA modification could provide novel insight into the mechanisms underlying the development and tumorigenesis of GC. The APA‐RNA and m^7^G‐RNA modifications were used to develop a prognostic scoring system in order to fully understand the correlation between these two modification patterns and GC prognosis.

The transcriptome data from 2412 GC samples were integrated into this investigation to conduct a comprehensive analysis of the biological patterns resulting from APA‐RNA and m^7^G‐RNA modifications. We detected two unique patterns of RNA modification and discovered that they were associated significantly with the cell cycle, KRAS, TGF‐β, and other signaling pathways related to cancer. Following that, we developed an RNA modification model (RMscore) that allowed us to measure the effectiveness of various alteration patterns in each GC patient. We discovered that the distribution of the RMscore was comparable with the ACRG molecular subtype, implying that this scoring method based on APA‐RNA and m^7^G RNA modifications plays a major role in determining GC prognosis.

## 2 METHODS

### 2.1 Dataset selection and data preprocessing

**FIGURE 1:**
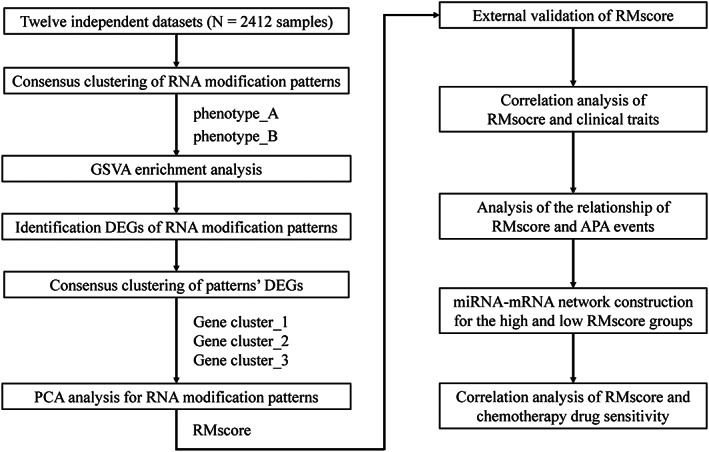
Flow chart of the study.

Figure 1 depicts the workflow for this investigation. We conducted a systematic search of public databases for GC‐related array datasets and chose GC microarray data published since 2010. Datasets with less than 50 samples and samples without survival information were excluded. We selected a total of 11 GC datasets from the Gene Expression Omnibus (GEO) (https://www.ncbi.nlm.nih.gov/geo/) for this study: GSSE100935, GSE15459, GSE26253, GSE26942, GSE34942, GSE35809, GSE51105, GSE54129, GSE57303, GSE62254, and GSE84437 (Table S1). One dataset was from The Cancer Genome Atlas (TCGA) (https://portal.gdc.cancer.gov/repository): TCGA‐STAD. Of the total 2412 samples, 2037 were from GEO, and the remaining 375 were from TCGA. All raw data in the GEO database were microarray data processed on Affymetrix and Illumina. The raw data retrieved from the Affymetrix platform were processed utilizing the RMA algorithm of the “affy” package in R for background adjustment and normalization.^13^ We downloaded both the Counts data and FPKM data of TCGA‐STAD for further analysis. In order to perform differential expression analysis, counts data were used, and the FPKM values were converted into TPM values that are more closely related to the data distribution obtained from microarray analysis and more consistent between samples examined on various platforms.^14^ Table S1 contains a summary of the information on the data that was obtained. The “ComBat” algorithm of the “sva” package in R was employed to eliminate the effects of batch between various cohorts.^15^ Additionally, relevant clinical data were extracted and manually arranged by direct download from the TCGA and GEO‐related websites or via a search of the reported original publications.

### 2.2 Consensus clustering based on APA and m^7^G modifications

The unsupervised clustering algorithm was used to perform a combined assessment on several datasets for the 20 marker genes in order to detect RNA modification patterns and classify samples for further investigation. Clustering consensus is a technique for computing the clustering consensus and evaluating its stability via repeated subsampling and clustering. It produces the clustering numbers that are the most closely related to the data matrix.^16^, ^17^, ^18^ We utilized the “ConsensusClusterPlus” package to conduct consensus clustering on the gene expression matrix of 2037 GEO samples, and 1000 iterations were carried out to confirm the classification's stability.

### 2.3 Functional annotation and gene set variation analysis (GSVA)

Unsupervised and nonparametric techniques were used to conduct GSVA to detect variations in the enrichment of pathways and biological processes among RNA modification patterns. GSVA was performed using the “GSVA” package in R.^19^ The gene sets “h.all.v7.3.symbols” and “c2.cp.kegg.v7.3.symbols” were downloaded from the MSigDB database for GSVA (http://www.gsea‐msigdb.org/gsea/downloads.jsp). Gene Ontology (GO) and Kyoto Encyclopedia of Genes and Genomes (KEGG) pathway studies were carried out with the help of the “clusterProfiler” package. A 0.05 (FDR) level of significance was considered to be statistically significant.

### 2.4 Differentially expressed genes (DEGs) between the RNA modification clusters

Following consensus clustering, the samples with different RNA modification patterns were classified into phenotype_A and phenotype_B. We utilized the R package “limma” to detect DEGs associated with phenotypes between the two clusters. This software analyses gene expression alteration using P‐values and t‐statistics obtained using Empirical Bayes estimation in a linear model.^20^ Adjustment of the P‐values was performed utilizing “Benjamini and Hochberg” approach. As a threshold for statistical significance, we used an adjusted *p* < 0.05 and |log FC| more than 1 (absolute value of log 2‐fold change).

### 2.5 Construction of a prognostic model using principal component analysis (PCA)

First, we repeated the consensus clustering on 56 DEGs related to phenotype from the 2037 samples to detect gene clusters associated with GC patients. As a result, we obtained three gene clusters for further analysis. Then, using a univariate Cox regression model, we evaluated the DEGs to determine which genes are related to the GC prognosis. Forty‐one significant genes were identified during this investigation (*p* < 0.001). Next, we performed Boruta feature selection using random forest machine learning using the “Boruta” package in R.^21^ All 41 genes were chosen as feature genes to construct a PCA scoring system. Most differences in the dimensional characteristics are preserved using PCA, one of the most extensively employed algorithms for data dimensionality reduction. Following the determination of each sample's principal component coefficient, we employed a procedure comparable to the analysis of gene–gene interaction^22^, ^23^ to get the RMscore for each sample:

$$\text{RMscore}=\sum \left(\mathrm{PC}1_{i}+\mathrm{PC}2_{i}\right)$$

where i means the RNA modification feature genes.

### 2.6 Association of *RM*score with posttranscriptional events

APA‐mediated 3' UTR shortening is a characteristic of the majority of cancer cells.^24^ To measure the abundance of APA events, we used a published algorithm termed “dynamic analysis of alternative polyadenylation from RNA‐seq” (DaPars). DaPars identified the abundance of APA events and calculated the “percentage distal‐polyA‐site usage index” (PDUI), which has the capability of quantifying the 3' UTR shortening or lengthening for each transcript.^25^ All the PDUI data were downloaded from The Cancer 3′ UTR Atlas (TC3A, http://tc3a.org).^26^ To compare the difference between groups with a high and a low RMscore, we used the “limma” package in R. An adjusted *p* < 0.05 and PDUI|log2 FC| > 0.12 were considered as a threshold for statistical significance.

Generally, the miRNA target sites are located in 3' UTRs.^10^ To evaluate the correlation between RMscore and miRNA, we conducted a series of analyses. First, miRNA and mRNA expression data of the GC matrix were downloaded from TCGA. Differential analysis of miRNA and mRNA between the groups with the high and low RMscore, was performed using the “limma” package. The anticipated miRNA target genes were paired with the findings of the differential analysis to generate an mRNA‐miRNA network employing DIANA TOOLS.^27^ KEGG enrichment analysis was employed to enrich the targeted signaling pathways of differentially expressed miRNAs. An adjusted *p* < 0.05 and |log FC| > 1 were considered as a threshold for statistical significance.

### 2.7 Association of RMscore with drug sensitivity and immune infiltration

For drug sensitivity analysis, we collected the drug response data from the Genomics of Drug Sensitivity in Cancer (GDSC, https://www.cancerrxgene.org/) database.^28^ To compare the sensitivity to chemotherapeutic drugs in the high and low RMscore groups, the “pRRophetic” package was used in R.^29^ We analyzed the relationship between RMscore and drug sensitivity using the Spearman method. We utilized CIBERSORT to evaluate the immune infiltration status of 300 patients from the GSE62254 dataset in order to determine whether the RMscore had an effect on immune cell infiltration. CIBERSORT is a linear support vector regression (SVR)‐based deconvolution algorithm that estimates the relative proportion of 22 immune cell types in cancer tissues.^30^

### 2.8 Statistical analysis

The Shapiro–Wilk normality analysis was conducted to assess the variables' normality. Continuous variables were compared between two groups utilizing the Mann–Whitney *U* test and unpaired Student *t*‐test for parametric and nonparametric data, respectively. We employed the parametric one‐way ANOVA or the nonparametric Kruskal–Wallis test for comparisons between more than two groups. The “survival” and “survminer” packages were utilized to produce survival curves employing Kaplan–Meier analysis, and the cutoff values were established using the “surv_cutpoint” function in the packages. The receiver operating characteristic (ROC) curve was achieved with the help of the “pROC” software package. Cox regression analyses, both univariate and multivariate, were carried out with the “survival” package in order to compute the hazard ratios and determine the independent prognostic factors in the study. All statistical tests used were two‐sided, with a *p* < 0.05 considered statistically significant. All of the statistical results were evaluated utilizing SPSS (version 26.0), R (version 3.6.2), and GraphPad Prism (version 9.0).

## 3 RESULTS

### 3.1 Landscape of genetic alterations of APA‐RNA and m^7^G‐RNA modification regulators in GC

**FIGURE 2:**
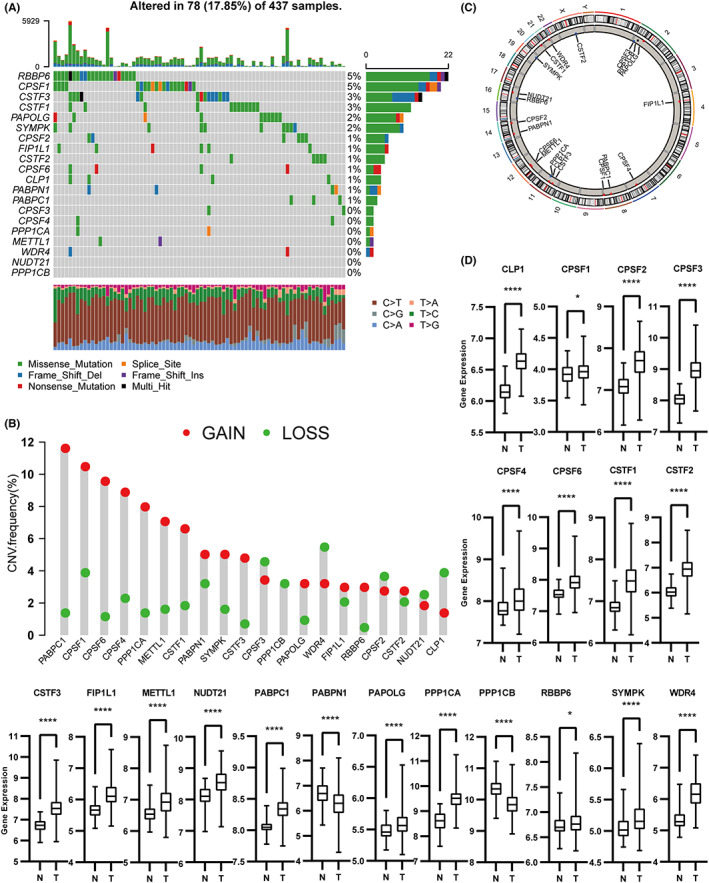
Landscape of genetic mutation and expression of marker genes in gastric cancer. (A) The mutation frequency of 20 marker genes of APA‐RNA and m^7^G‐RNA modification in 437 patients with gastric cancer from TCGA‐STAD cohort. The upper barplot indicated TMB per patient, whereas the right barplot showed the mutation frequency of each gene mutation type. The stacked barplot below showed the fraction of nucleotide base conversions in each patient. (B) The CNV alteration frequency of marker genes. The red dot represents CNV gain, and the green dot represents CNV loss. (C) The position of CNV changes in marker genes across 23 chromosomes. (D) The expression of 20 marker genes between normal and tumor samples. The asterisks represented the statistical *p*‐value (*p < 0.05, **p < 0.01, ***p < 0.001, ****p < 0.0001).

We identified 20 RNA modification‐related genes based on the published data in this study.^6^, ^7^, ^10^, ^11^ To explore the genetic mutation of these genes in GC, we assessed somatic mutations and the incidence of copy number variations (CNVs). Of the 437 available samples from TCGA, 78 (17.85%) underwent mutations of RNA modification. We discovered that the most frequently mutated gene was RBBP6, followed by CPSF1 (Figure 2A). Seven genes did not show any mutations in GC samples including CPSF3/4, PPP1CA/B, NUDT21, and m^7^G‐related genes (METTL4 and WDR4). Further analysis of CNV alteration frequency showed that PABPC1 and CPSF1/4/6 had a high frequency of CNV amplification, whereas WDR4, CPSF3, and CLP1 mainly showed CNV depletion (Figure 2B). Figure 2C depicts the chromosomal location of CNV changes in the modification regulators. We compared the gene expression in relation to APA‐RNA and m^7^G‐RNA modifications between normal and GC patients to determine whether the genetic mutations affected the related gene expression. As shown in Figure 2D, we found that RNA modification genes with a high frequency of CNV gain were overexpressed in GC patients, implying that CNVs could be a potential contributor to the regulation of the expression of RNA modification‐related genes. However, some genes with a high frequency of CNV loss showed high expression in GC patients too. Furthermore, we investigated the correlation between CNV loss and the expression of five such genes (Figure S1A–E). In the four groups including normal, CNV loss, CNV gain, and no CNV groups, we discovered that the expression of mRNA of the majority of genes in the CNV loss group (excluding CPSF3) was significantly reduced or comparable to (no significance) that of the normal group. Additionally, the CNV gain group expressed these genes more than the other groups. However, tumorigenesis is a multistep and complex dynamic process.^31^ One study suggested that CNV frequency does not completely correlate with the protein levels,^32^ indicating that CNVs are but one among the many regulatory factors of tumorigenesis and can partly explain the differential expression of RNA modification‐related genes.

The landscape of genetic alterations revealed the distribution and frequency of the mutations of RNA modification‐related genes and allowed us to compare the differential expression of genes between the four alteration groups in GC.

### 3.2 Consensus clustering depending on marker genes and functional enrichment analysis using GSVA

**FIGURE 3:**
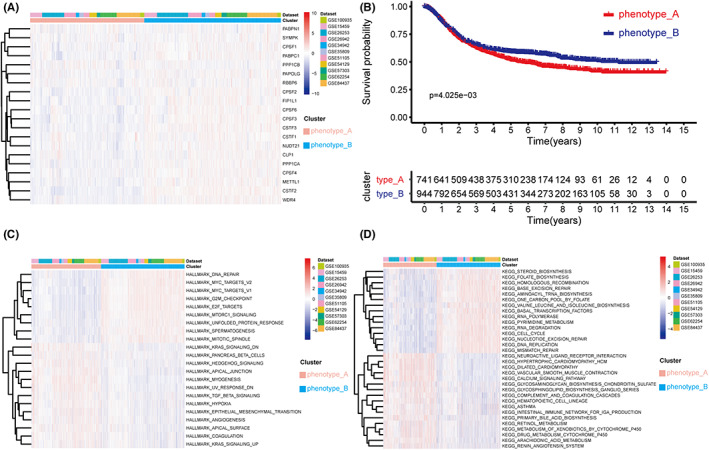
Patterns of RNA modification and GSVA functional enrichment analysis. (A) Unsupervised consensus clustering for 2037 GEO gastric cancer patients of 11 datasets. (B) Kaplan–Meier curve revealed a substantial difference between two RNA modification clusters. The red represented phenotype_A and the blue represented phenotype_B. (C–D) GSVA enrichment analysis using “hallmark” and “KEGG” gene sets.

Consensus clustering was utilized to characterize the RNA modification patterns using the expression data for the 20 recognized marker genes. Finally, we found two unique groups, phenotype_A and phenotype_B, which contained 927 and 1110 samples, respectively (Figure S2A–C; Table S2). The heatmap (Figure 3A) shows that high gene expression focused on phenotype_B, whereas low gene expression focused on phenotype_A. Kaplan–Meier investigation revealed that phenotype_B had a better prognosis than phenotype_A (Figure 3B, *p* = 4.025e‐03). Then, GSVA was carried out to compare the pathway enrichment and biological process between phenotype_A and phenotype_B utilizing gene sets (“hallmark” and “KEGG”). In most published papers, hallmark gene sets summarized substantially well‐defined biological processes and showed excellent consistency.^33^ As illustrated in Figure 3C, phenotype_A was highly enriched in several cancer‐associated pathways, including KRAS, Hedgehog, TGF‐β, hypoxia, angiogenesis signaling pathways, and epithelial‐mesenchymal transition (EMT), suggesting that phenotype_A is related to tumor invasion and metastasis. Phenotype_B was markedly enriched in cell cycle‐related pathways, including DNA repair, G2M checkpoint, UNFOLDED protein response, E2F targets, MYC targets v2, MTORC1 signaling, MYC targets v1, spermatogenesis, and MITOTIC spindle assembly (Figure 3C, adjusted *p* < 0.05). To identify the higher proliferation ability of phenotype_B, we compared MKI67 expression between the two clusters and discovered that phenotype_B had considerably greater MKI67 expression (Figure S2D). KEGG pathway enrichment analysis of the two clusters mainly focused on metabolism‐ and proliferation‐related pathways, including lipid metabolism, nucleic acid metabolism, DNA replication, and mismatch repair pathways. Thus, the results of GSVA revealed that phenotype_A is highly related to metastasis and tumor invasion, whereas phenotype_B is significantly associated with cell proliferation (Figure 3D).

### 3.3 Construction of a signature for RNA modification

**FIGURE 4:**
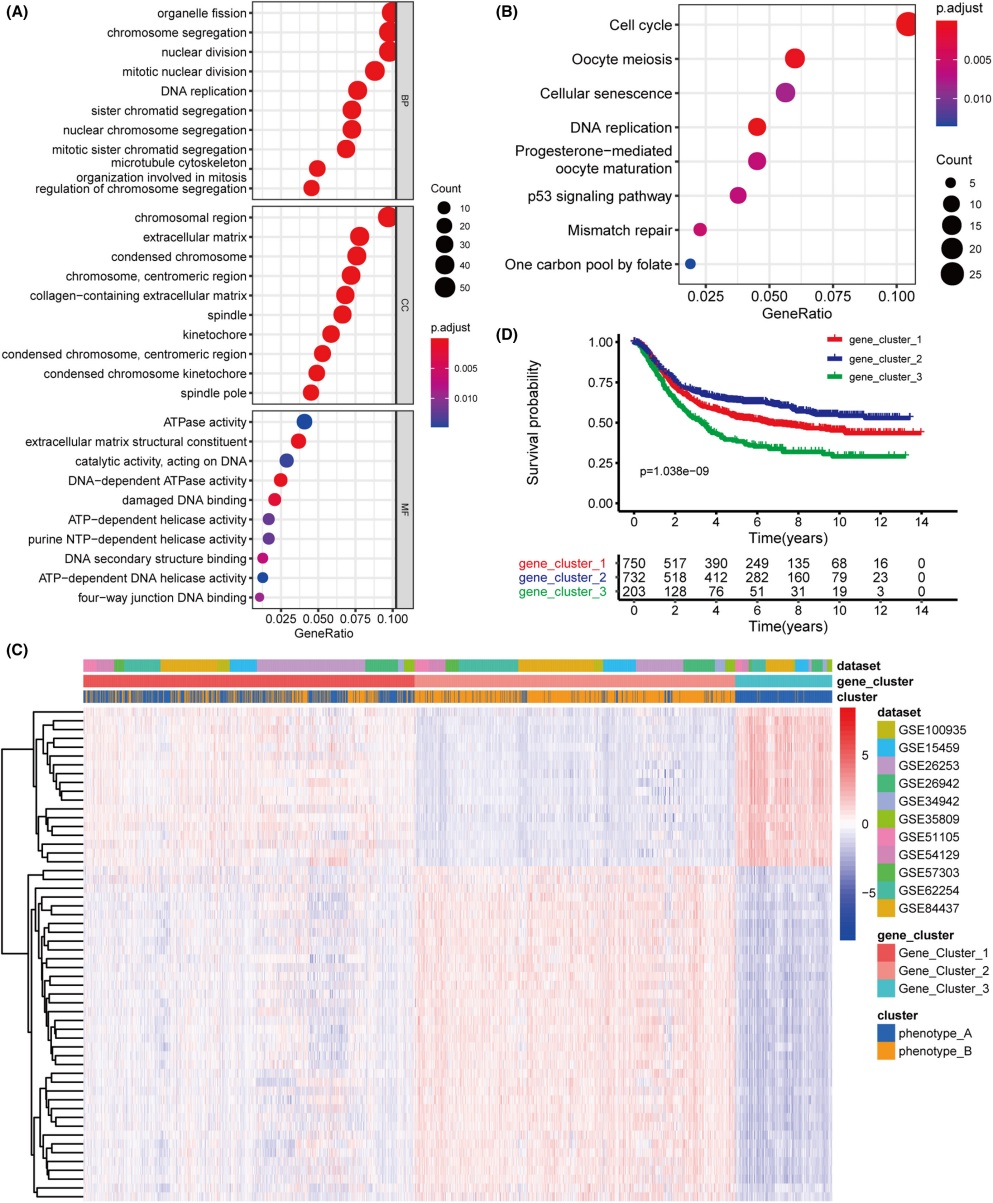
Construction of RMscore model. (A–B) GO and KEGG enrichment analysis of DEGs between two clusters abovementioned. (C) Unsupervised consensus clustering of DEGs using expression data was used to divide patients into three groups, called Gene clusters 1–3. (D) Kaplan–Meier curves for the patients of three Gene clusters.

The differential analysis of the two RNA modification patterns revealed their potential biological functions. We identified 566 DEGs with |log FC| > 0.585 and an adjusted *p* < 0.05 is used as the threshold for significance (Figure S2E). Further functional enrichment analysis demonstrated that DEGs were enriched for biological processes related to cell proliferation and cell cycle (Table S3). Except for cell replication‐related pathways, KEGG pathway enrichment analysis focused on mismatch repair, p53 signaling pathway, and cellular senescence (Table S4; Figure 4A,B). To detect the genes related to the prognosis in GC, the DEGs were selected with |log FC| > 1 to perform univariate Cox regression (*n* = 56, Table S5). The 41 prognostic genes are shown in Figure S2F and Table S6 (adjusted *p* < 0.001). We also discovered genomic subtypes based on the prognostic genes using the consensus clustering method (Figure S3A–C). Three subtypes were identified: gene_cluster_1, gene_cluster_2, and gene _cluster_3. These subtypes had 901, 872, and 264 samples, respectively (Figure 4C; Table S2). Additionally, we discovered that the majority of samples in gene _cluster_1 and gene_cluster_3 were classed as phenotype_A, while those in gene_cluster_2 were closely associated with phenotype_B. The abovementioned association was also confirmed by Kaplan–Meier analysis (Figure 4D). A better prognosis was observed in the patients in gene_cluster_2 when compared to the patients in the other two clusters (*p* = 1.038e‐09), which was comparable with the pattern of a better prognosis being observed in patients in phenotype_B.

The above results proved the effectiveness and stability of APA‐RNA and m^7^G‐RNA modification patterns.

### 3.4 Further survival analysis and external validation of the RMscore model

**FIGURE 5:**
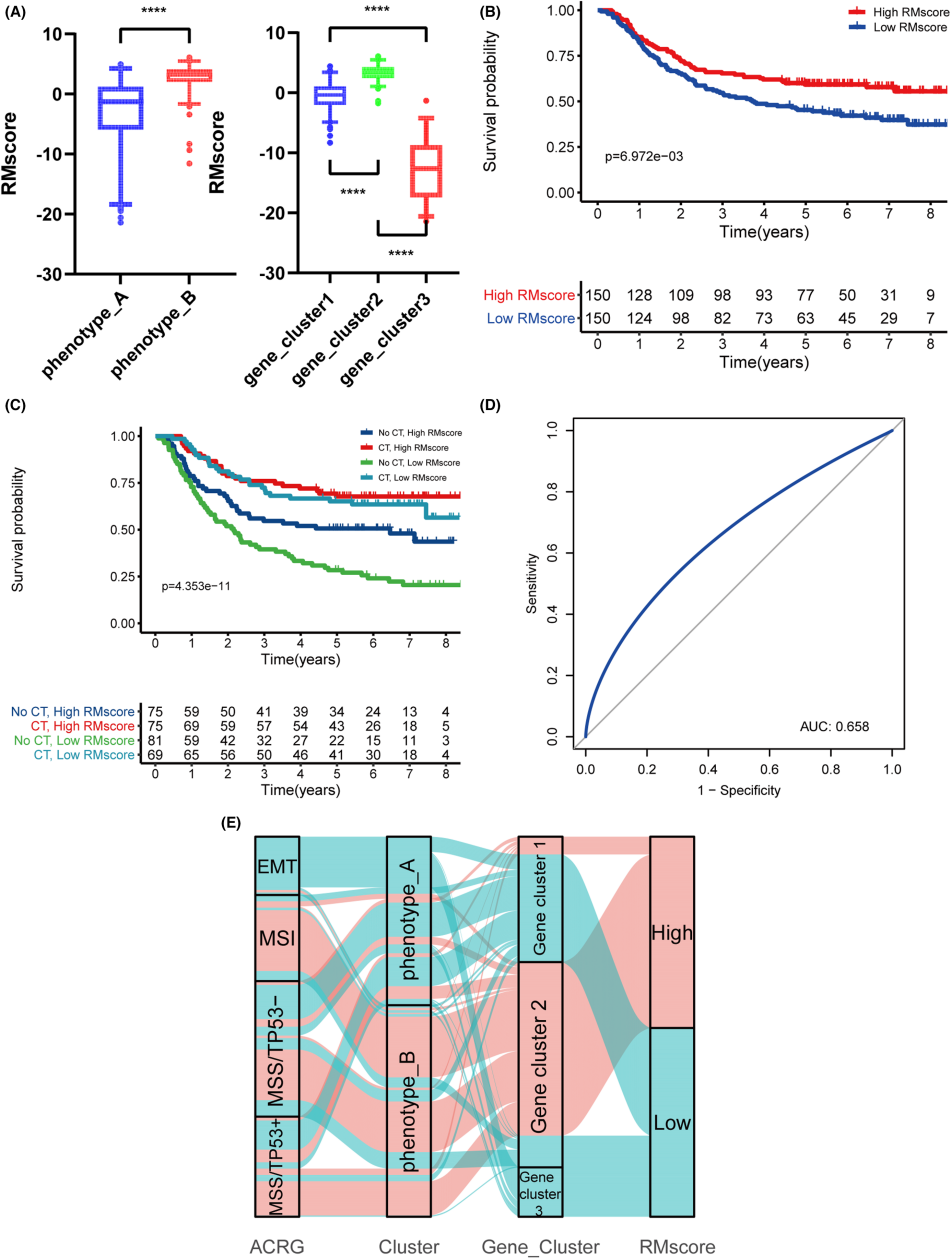
The feature of RMscore characteristic signature. (A) Distribution of RMscore in RNA modification patterns. (B) Kaplan–Meier curves for patient groups with a high and a low RMscore in the ACRG cohort. (C) Kaplan–Meier curves for ACRG patients stratified by adjuvant chemotherapy and RMscore. (D) The ROC curve for the RMscore model. (E). Alluvial diagram of ACRG molecular subtypes in groups with different Clusters, Gene clusters, and RMscore.

Furthermore, we developed a scoring system based on the PCA approach for quantifying the APA‐RNA and m^7^G‐RNA modification patterns of individual GC patients (Table S7). The model was based on the ACRG cohort and named the RNA modification score (RMscore). We found that phenotype_B had a much higher RMscore than phenotype_A, and gene_cluster_2 demonstrated a significantly higher RMscore than gene_ cluster_1 and gene_cluster_3 (Figure 5A, *p* < 0.01). Kaplan–Meier study demonstrated that patients with a high RMscore had a considerably better prognosis in comparison with patients with a low RMscore (Figure 5B, *p* = 6.972e‐03). Furthermore, survival analysis was conducted on patients in the ACRG cohort who had adjuvant chemotherapy to assess the prognostic analytic effect of RMscore in patients who took or did not take adjuvant chemotherapy. We revealed that among both patients who received chemotherapy and those who did not, patients with a high RMscore showed higher overall survival (Figure 5C, *p* = 4.353e‐11), and the area under the curve (AUC) of the ROC was 0.658 (Figure 5D). To identify the association of the ACRG subtype with the RMscore, we traced each sample from the ACRG subtype to the RMscore. As shown in Figure 5E, almost all patients with the EMT subtype were classified into phenotype_A and then into gene_cluster_1 or gene_cluster_3, and finally they were assigned a low RMscore, which was consistent with poor prognosis in these groups. However, most patients with the MSI subtype were assigned a high RMscore, and they had a better prognosis.^34^

Following that, we conducted uni and multivariate Cox regression analyses to assess whether the RMscore could be employed as an independent prognostic factor for GC. According to the findings, the RMscore is a strong independent prognostic factor. (Figure S3D–E, HR 0.960 [0.935–0.985], *p* < 0.01).

**FIGURE 6:**
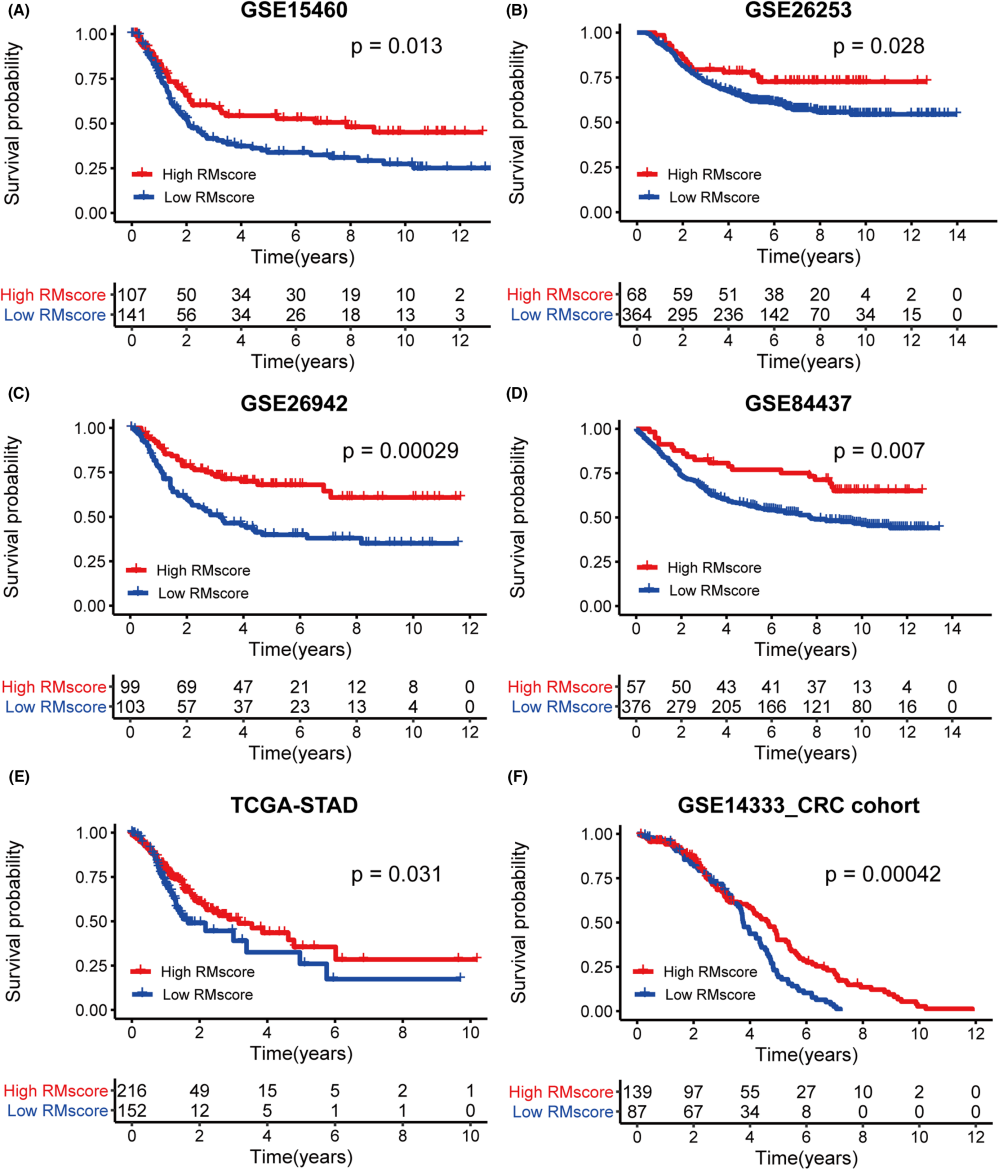
The RMscore model's external validation. (A–F) Kaplan–Meier curves for patients with high and low RMscores in four GEO datasets (GSE15460, GSE26253, GSE26942, and GSE84437), the TCGA‐STAD cohort, and a CRC cohort (GSE14333).

In order to validate the stability of the RMscore model, we applied the RMscore signature established in the ACRG cohort to six external independent GEO or TCGA GC datasets. The findings of the validation examination demonstrated that patients with a higher RMscore had a better prognosw3sis, (Figure 6A–E, all *p* < 0.05). Next, we attempted to expand the application of this model to other cancers, such as colorectal cancer (CRC). Kaplan–Meier survival analysis indicated that CRC patients with a low RMscore had a poor prognosis (Figure 6F, *p* = 4.2e‐04). These results demonstrated that RMscore is an effective and stable model to explain APA‐RNA and m^7^G‐RNA modifications and can predict the prognosis of patients with gastrointestinal tumors, especially GC.

### 3.5 GSVA and clinical significance of the RMscore model

**FIGURE 7:**
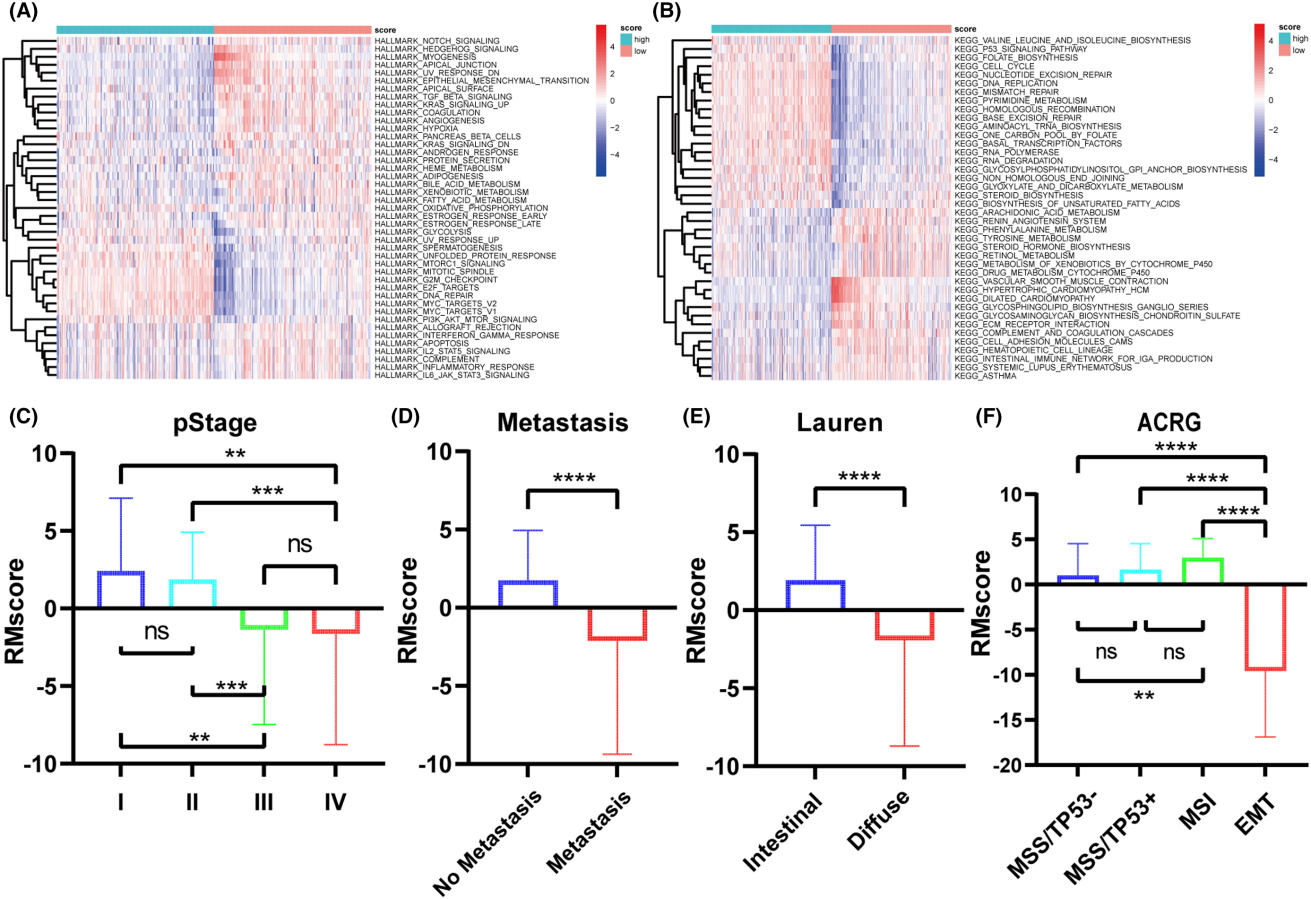
Clinical significance and GSVA functional enrichment of RMscore model. (A–B) GSVA study of patients with a high or a low RMscore (A, Hallmark gene sets; B, KEGG gene sets). (C–F) The association of clinical traits and RMscore (C, pStage; D, metastasis; E, Lauren subtype; F, ACRG molecular subtype).

Analysis of functional enrichment demonstrated that the low RMscore group was enriched in Notch, hedgehog, TGF‐β, EMT, PI3K‐AKT–MTOR, and KRAS signaling pathways, and the high RMscore group was enriched in cell proliferation‐associated pathways such as DNA repair, UNFOLDED protein response, MYC targets v2, E2F targets, MTORC1 signaling, MYC targets v1, G2M checkpoint, spermatogenesis, and MITOTIC spindle assembly pathways (Figure 7A,B). The enrichment results were similar to those of phenotype_B, thus confirming the reliability of the RMscore model.

To investigate the clinical importance of RMscore, we examined the relation between RMscore and clinical traits, including Lauren subtype, pathological stage, ACRG molecular subtype, and metastasis (Figure 7C–F). We found that the RMscore decreased with an increase in the GC stage, which translates to a worse prognosis. The RMscore of patients with metastasis was also significantly reduced. Similarly, as for patients with the Lauren subtype, the RMscore of patients with the diffuse subtype was considerably lower than that of patients with the intestinal subtype. The ACRG molecular subtype was one of the most commonly used molecular subtypes of GC.^34^ Consistent with the ACRG cohort's overall survival study, patients with the MSI subtype had a relatively high RMscore, followed by MSS/TP53+, MSS/TP53‐, and lastly the EMT subtype. The correlation between RMscore and clinical characteristics showed that RMscore is closely associated with the clinical stage and molecular subtype of GC patients, and the trends of prognosis prediction were consistent, which further confirms the clinical significance of RMscore.

### 3.6 Association of RMscore with drug sensitivity and immune infiltration

**FIGURE 8:**
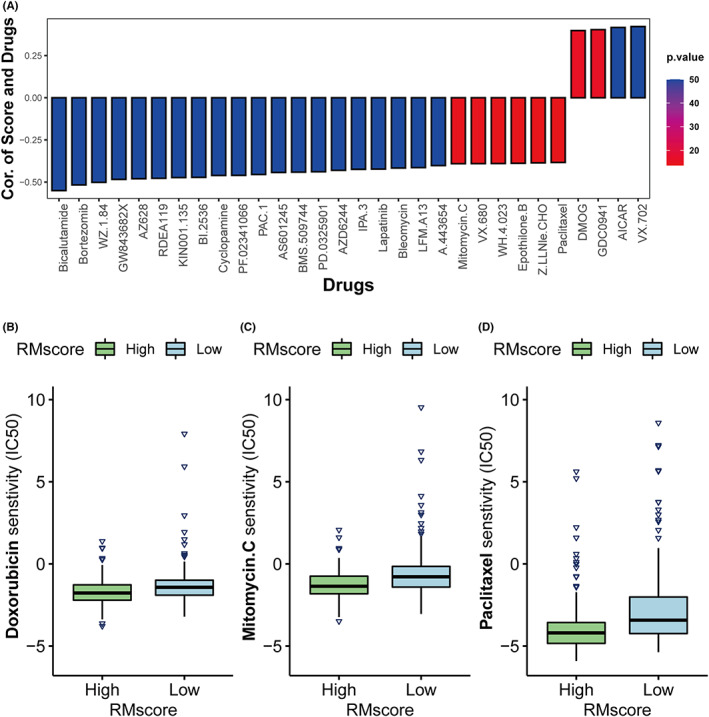
The relationship of RMscore and drug sensitivity. (A) The relationship between RMscore and drug sensitivity determined using the Spearman method. The barplot represented correlation coefficient. The right column represented the *p*‐value. (B–D) Comparison of three representative drugs in high‐ and low‐ RMscore groups (B, Doxorubicin; C, Mitomycin C; D, Paclitaxel).

Research on drug sensitivity linked the drug response to possible biological effects through analysis of gene expression profiles. To acquire a better understanding of the RMscore's effect on the response of drugs, we examined the correlation between the RMscore and data on the response of drugs in cancer cell lines obtained from the GDSC database. The Spearman correlation analysis was performed using the “pRRophetic” package, which contained 138 drugs for analysis (Table S8). Totally, 41 drugs were identified (|cor.| > 0.35, adjusted *p* < 0.05), and we showed the correlation and significance of 30 drugs with the highest correlation coefficients (Figure 8A), including the proteasome inhibitor bortezomib (cor. = −0.52), the Hedgehog inhibitor cyclopamine (cor. = −0.46), the EGFR inhibitor lapatinib (cor. = −0.42), and others. Notably, we focused on three drugs used in clinical GC chemotherapy: doxorubicin (cor. = −0.35), mitomycin C (cor. = −0.39), and paclitaxel (cor. = −0.38). We compared the IC50 (an indicator of drug sensitivity) of these three drugs between patients with high and low RMscore using the Mann–Whitney *U* test (Table S9). The high RMscore patients showed significantly increased sensitivity to these three drugs (Figure 8B–D). These findings suggested that RNA modification patterns are related to drug sensitivity. And RMscore might have potential applications in the development of novel chemotherapeutic drugs.

In terms of immunological infiltration, we observed that resting memory CD4+ T cells (*p* = 0.002), M2 macrophages (*p* = 0.047), and resting dendritic cells (*p* < 0.001) were considerably lower in a group with the high RMscore than in the group with the low RMscore. On the contrary, the activated memory CD4+ T cells (*p* < 0.001), M1 macrophages (*p* = 0.032), activated dendritic cells (*p* = 0.007), and neutrophils (*p* = 0.004) in the group with the high RMscore were considerably more than those in the group with the low RMscore (Figure S4). Immune infiltration study revealed that the RNA modification patterns were related to both memory CD4+ T cells and innate immune cells, including macrophages, eosinophils, dendritic cells, and neutrophils.

### 3.7 RNA modification model involved in posttranscriptional regulation

**FIGURE 9:**
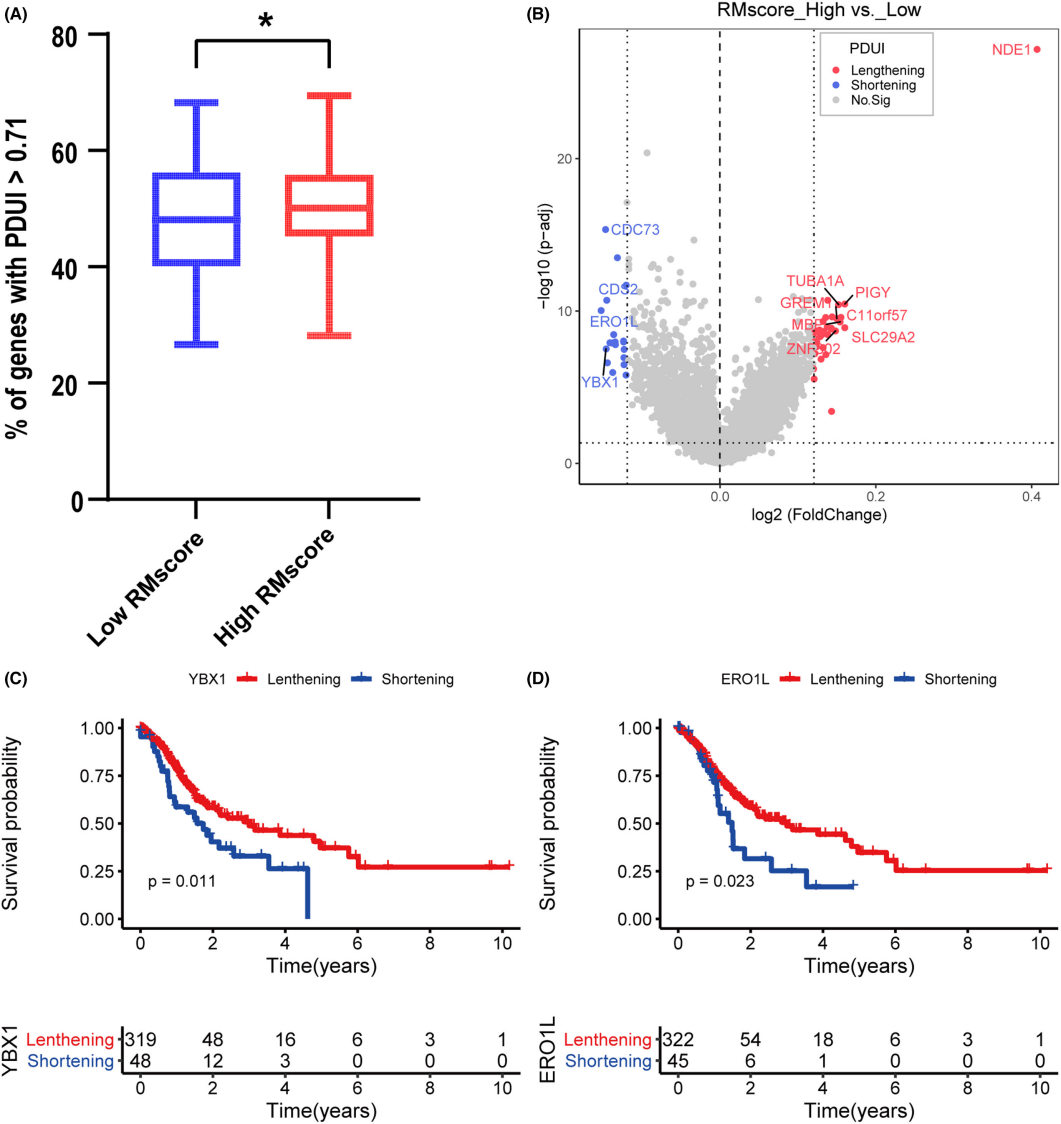
Posttranscriptional characteristics associated with the RMscore. (A) Comparison of percentage of transcripts with PDUI greater than 0.71 divided by total number of APA events between high‐ and low‐RMscore groups. (B) The differential analysis in PDUI between the groups with high and low RMscore. (C–D) Kaplan–Meier curves for PDUI lengthening and shortening of YBX1 and ERO1L.

Given that APA events are essential for posttranscriptional regulation (including pre‐mRNA maturity, slicing, and cellular location), we assessed the relationship between RMscore and PDUI. We set PDUI > 0.71 (median value of all samples) as the cutoff for relatively longer 3' UTR isoforms. The percentage of long 3' UTR genes in the group with a high RMscore was considerably greater than that in the group with a low RMscore (Figure 9A, *p* = 0.022). We identified the differential PDUI profile between high RMscore and low RMscore groups and compared the prognosis to determine the association of the length of 3' UTR and overall survival of GC patients (Figure 9B). The results revealed that patients with a high score had transcripts with a longer 3' UTR and vice versa, consistent with the finding of a previous study.^35^ YBX1 was a versatile RNA‐binding protein that was highly overexpressed in multiple cancer types,^36^, ^37^ and NDE1 and ERO1L were the two most DEGs. The Kaplan–Meier curve revealed that the shortening of YBX1 and ERO1L was related to poor prognosis (Figure 9C,D, *p* = 0.011 and 0.023, respectively). However, consistent with a previous study,^11^ NDE1 displayed lengthening of 3' UTR. We propose a possibility that in the low RMscore group, genes (such as YBX1) with shortening of 3' UTR might not be targeted by miRNA, resulting in the dysregulation of biological processes and contributing to tumorigenesis.

**FIGURE 10:**
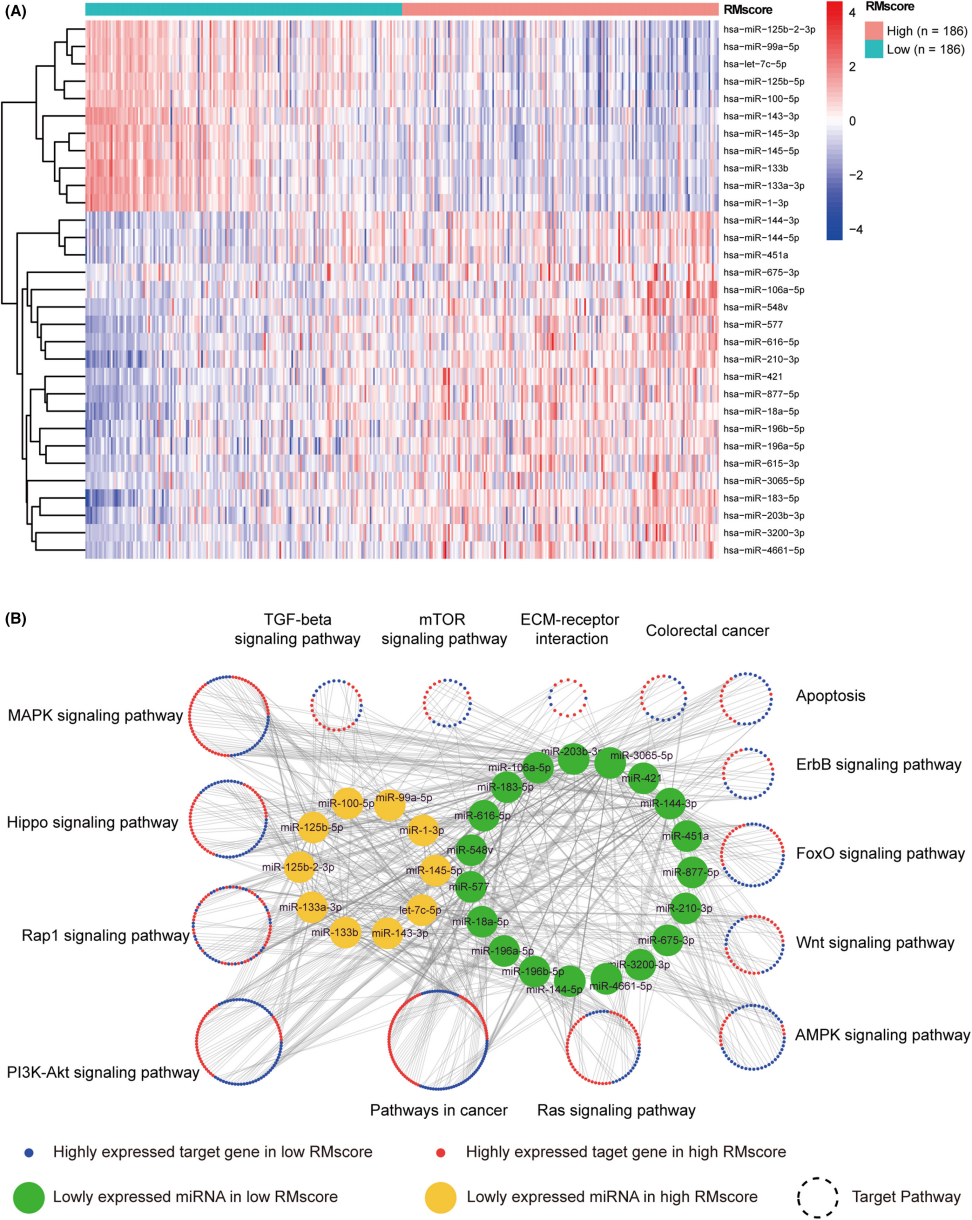
The miRNA‐mRNA network as determined by the RMscore. (A) Differential analysis of miRNA between the groups with high and low RMscore. (B) The miRNA‐mRNA network as determined by the RMscore and the signaling pathways of the target genes.

Shortening of the 3' UTR might increase the loss of miRNA‐binding targets. Hence, we thought that RMscore may reveal any change in the regulation of the miRNA‐mRNA network. We used differential expression analysis to determine the expression of miRNA and mRNA and 31 miRNAs were identified (Figure 10A, |log FC| > 1, *p*‐value [adjusted] less than 0.05). To determine the functional features of miRNA and target genes, we constructed the miRNA‐mRNA network that showed the differentially expressed miRNA and mRNA between the high RMscore and low RMscore groups (Figure 10B). The miRNAs that were differentially expressed between the groups with the high and low RMscore were highly enriched in several signaling pathways related to cancer, such as TGF‐β, mTOR, PI3K‐Akt, AMPK, and Wnt signaling pathways. Upregulated miRNAs were determined to be enriched in the AMPK, MAPK, and apoptosis pathways in the high RMscore group, whereas downregulated miRNAs were determined to be enriched in the Hippo, PI3K‐Akt, and Rap1 signaling pathways. These results indicated that RMscore is closely related to posttranscriptional regulation.

## 4 DISCUSSION

This study mainly observed the prognosis of GC patients from the perspective of APA‐RNA and m^7^G‐RNA modifications, and identified the important role of the RMscore in survival trends, clinicopathological behaviors, anticancer drug sensitivity, and potential regulatory pathways in GC. First, we classified the modification patterns in accordance with the expression profiles of 20 marker genes into two phenotype clusters. Following that, we used 41 DEGs to identify three genomic subtypes. The RMscore model was then developed in order to measure RNA modifications and anticipate the prognosis of GC patients. Correlation analysis showed that the higher the RNA modification score, the lower is the malignancy of the tumor, and the better is the prognosis of the patient. ACRG subtype analysis showed that there are significant differences in RMscore in different molecular subtypes. Patients with EMT subtypes have low RMscore and poor prognosis; those with MSI subtypes showed the opposite result. Our research reveals the important role of APA‐RNA and m^7^G‐RNA modification in the biological behavior, survival, prognosis, and drug sensitivity of GC.

Globally, the clustering method and RMscore model demonstrated accuracy and consistency. The samples with the low RMscore were largely connected with phenotype_A as well as gene_cluster_1 and gene_cluster_3, according to the results of the analysis. When the RMscore was low, the prognosis for GC was even worse. The findings of the functional analysis demonstrated that several signaling pathways related to cancer, including Hedgehog, KRAS, and TGF‐β signaling pathways, were activated in samples with a low RMscore. The KRAS signaling pathway is activated not just by conventional mutations, but also by high‐level KRAS gene amplification.^38^ The KRAS signaling system is related to resistance to targeted chemotherapies and is related to the development and progression of gastroesophageal cancers.^39^ In tumor cells, aberrant Hedgehog signaling results in self‐renewal, invasion, and proliferation. The Hedgehog signaling pathway was abnormally active in samples with a low RMscore, promoting the transformation of cancer stem cells, and carcinogenesis.^40^ TGF‐β functions as a two‐edged sword. TGF‐β signaling plays a critical function in the promotion and suppression of tumors. In this work, we determined that the low RMscore group activated the EMT pathway, and TGF‐β signaling has been shown to increase GC development by inducing EMT.^41^ Phenotype_B showed different functional biological processes mainly related to cell cycle signaling pathways. Samples with a low RMscore were enriched in cell proliferation‐related pathways including DNA repair, MYC targets v1, E2F targets, MYC targets v2, UNFOLDED protein response, G2M checkpoint, MTORC1 signaling, spermatogenesis, and MITOTIC spindle assembly pathways. These functional activities indicated that phenotype_B is correlated with hyperactive tumor cell proliferation ability. The cell proliferation antigen MKI67 is a widely used marker in tumor histopathology,^42^ and the high expression of MKI67 in phenotype_B confirmed the hyperactive proliferation activity. Consistently, we observed that the RMscore was much lower in advanced GC, implying that a low RMscore may promote the development of GC by activation of the aforementioned signaling pathways.

APA events may lead to the loss of miRNA‐binding sites in 3' UTR and interfere with miRNA regulation mechanisms, resulting in protein expression dysregulation and promoting their tumor growth or weakening their tumor suppressor effects.^43^ In this study, RNA modifications were found to affect the regulation of MAPK, PI3K‐Akt–mTOR, Ras, and other signaling pathways in the miRNA‐mRNA regulation network through the differential expression miRNAs, such as miR‐3065‐5P and miR‐144‐3p. Specifically, miR‐144‐3p expression was dramatically high in groups with high RMscore, and the target genes (also the DEGs between groups with the high and low RMscore) were highly enriched in the PI3K‐Akt–mTOR signaling pathway. The abnormal activation of this signaling pathway modulates autophagy, EMT, apoptosis, and metastasis in many types of cancers.^44^

Then, we investigated the therapeutic potential of RNA modifications in GC. Numerous signaling pathways are involved in tumor resistance, including PI3K‐Akt–mTOR and TGF‐β signaling pathways. The over‐activation of PI3K‐Akt–mTOR or TGF‐β signaling pathways has been reported as the underlying causative mechanism.^45^, ^46^ Paclitaxel, mitomycin C, and doxorubicin are effective chemotherapeutic drugs used in clinical practice.^47^ The high RMscore group had considerably greater drug sensitivity than the low RMscore group for these three drugs. These findings demonstrated that patients with a high RMscore might benefit from chemotherapy that targets the associated signaling pathways. RMscore might be a potential facilitator for the development of targeted chemotherapeutic drugs.

The widely recognized molecular classifications of GC mainly include TCGA molecular classification and ACRG molecular classification. Here, we compared the clinical information of patients between RMscore groups and ACRG subtypes. The molecular subtype based on the ACRG, which emphasizes GC heterogeneity, is composed of four molecular subtypes associated with unique molecular changes, disease prognosis, and progression.^34^ The MSI subtype exhibited the best prognosis in the ACRG analysis, followed by MSS/TP53‐ and MSS/TP53+, with the MSS/EMT subtype having the poorer prognosis.^34^ In accordance with the survival trends observed in the ACRG cohort, we discovered a similar distribution in the RMscore model; GC samples corresponding to the EMT subtype were significantly associated with a low RMscore and a poorer prognosis.

There was a classic study in 2017 that proposed three main cancer‐immune phenotypes: inflamed phenotype, immune desert phenotype, and immune excluded phenotype.^48^ However, we did not find a significant difference in the CD8+ T cells proportion in patients between groups with the high and low RMscore. Nevertheless, we determined that CD4+ memory T cells, macrophages, neutrophils, and dendritic cells play an essential function in the APA‐RNA and m^7^G‐RNA modification patterns in patients with GC. Increased infiltration of activated memory CD4+ T cells, neutrophils, and M1 macrophages was found in patients with a high RMscore, supporting prior results that these immune cells play an essential function in the microenvironment of the tumor. Activated memory CD4+ T cells can boost cognate memory CD8+ T cell expansion and improve tumor control.^49^, ^50^ The more tumor‐infiltrating neutrophils, the better is the prognosis of GC patients, and chemotherapy may be more sensitive.^51^

WHO classification and Lauren classification, two of the most commonly used GC classification techniques in clinical practice, without a quantitative score for each patient and have limited clinical usefulness. Preclinical applications of molecular subtype systems regarding the molecular mechanisms and clinical outcomes, including the ACRG and TCGA subtypes, are simple to implement in the preclinical setting and aid in clinical decision‐ making. Cancer‐immune phenotypes classify the tumor immunological signature into three subtypes. However, tumor immunity is complex and variable, necessitating a broader view of cancer immunity. The goal of this investigation was to evaluate the molecular function of APA‐RNA and m^7^G‐RNA modifications in GC. We assessed RNA modifications for each patient individually and constructed a scoring system that related the RMscore to GC patient prognosis.

Several limitations were identified in our investigation. First, considering individual GC heterogeneity, the findings of our investigation need to be validated further by a larger number of multicenter clinical studies. Second, we have examined the correlation between RMscore and immune infiltration. Regrettably, no significant differences in CD8+ lymphocyte levels were observed. According to the survival analysis in IMvigor210 and GSE78220 cohorts (anti‐PD‐1/L1 immunotherapy), the RMscore model failed to show consistent efficacy of immunotherapy in these two cohorts. The underlying relationship between RMscore and immunotherapy awaits follow‐up studies. Finally, our results have important implications for GC RNA modification, and the detailed molecular mechanisms need additional investigation to elucidate deeper relationships.

## 5 CONCLUSIONS

This work focused on the investigation of APA‐RNA and m^7^G‐RNA modifications in GC, and we developed the RMscore model based on the modification patterns in order to investigate the vast regulatory mechanisms by which RNA alterations affect carcinogenesis in greater detail. We established a correlation between the scoring system and clinical outcomes in GC patients and validated the model using numerous external datasets from the TCGA and GEO databases. This investigation has provided novel information about the role of RNA modifications in the development of GC.

## AUTHOR CONTRIBUTIONS

QC, YT, and HX: conception and design. QC, ZL, and YT: acquisition of data. QC, SP, and WA: analysis and interpretation of data. QC, ZL, and HX: writing and review of the manuscript. The final version of this work has been read and approved by all of the authors.

## FUNDING INFORMATION

This research was supported by the National Natural Science Foundation of China (No. 81772549), National Natural Science Foundation of China (No. 81572334).

## CONFLICT OF INTEREST

The authors state that they had no commercial or financial affiliations that may be considered a potential conflict of interest when conducting the research.

## Supporting information


Supplementary Figure 1

Supplementary Figure 2

Supplementary Figure 3

Supplementary Figure 4

Supplementary Table 1

Supplementary Table 2

Supplementary Table 3

Supplementary Table 4

Supplementary Table 5

Supplementary Table 6

Supplementary Table 7

Supplementary Table 8

Supplementary Table 9

## Data Availability

The data sets presented in this study can be found in online repositories. The names of the repository/repositories and accession number(s) can be found in the article.
