# Supplementary figures and images for: Characterization of RNA modifications in gastric cancer to identify prognosis‐relevant gene signatures
Source: Cancer Med. 2022 May 30;12(1):879–97. doi: 10.1002/cam4.4861 (PMC9844604; doi:10.1002/cam4.4861)

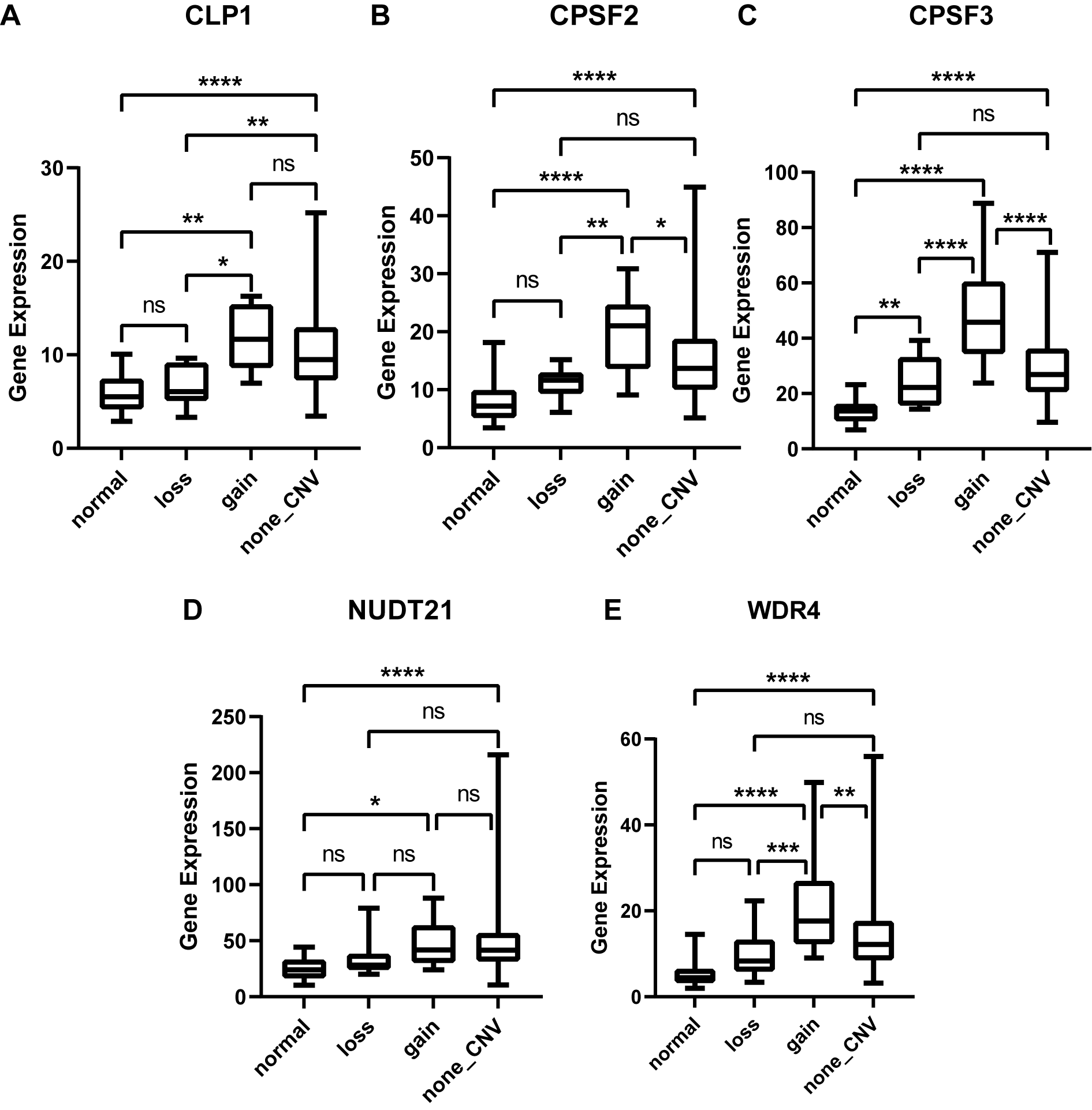

Supplement: Supplementary file 1 — Supplementary Figure 1 [file CAM4-12-879-s008.tif]

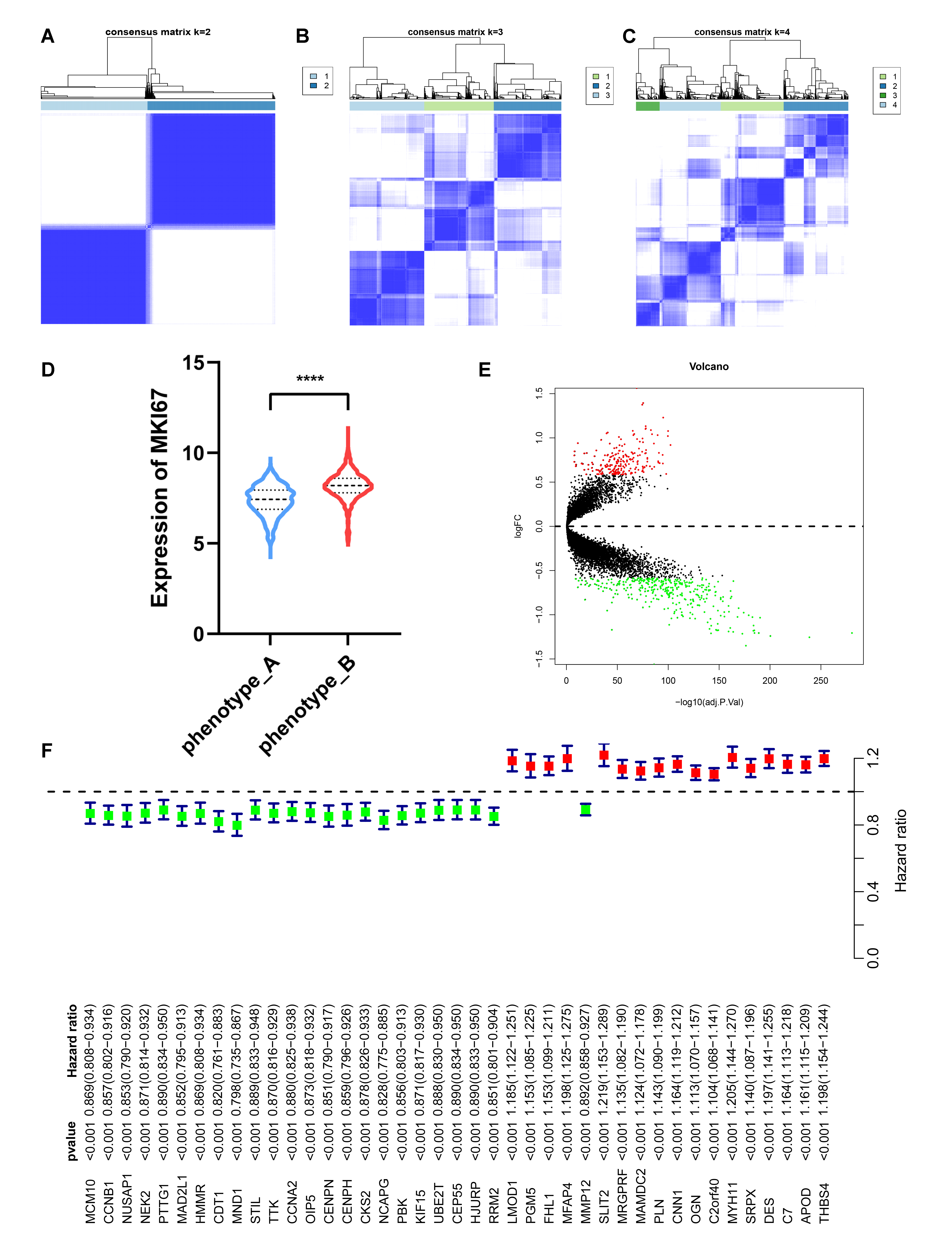

Supplement: Supplementary file 2 — Supplementary Figure 2 [file CAM4-12-879-s003.tif]

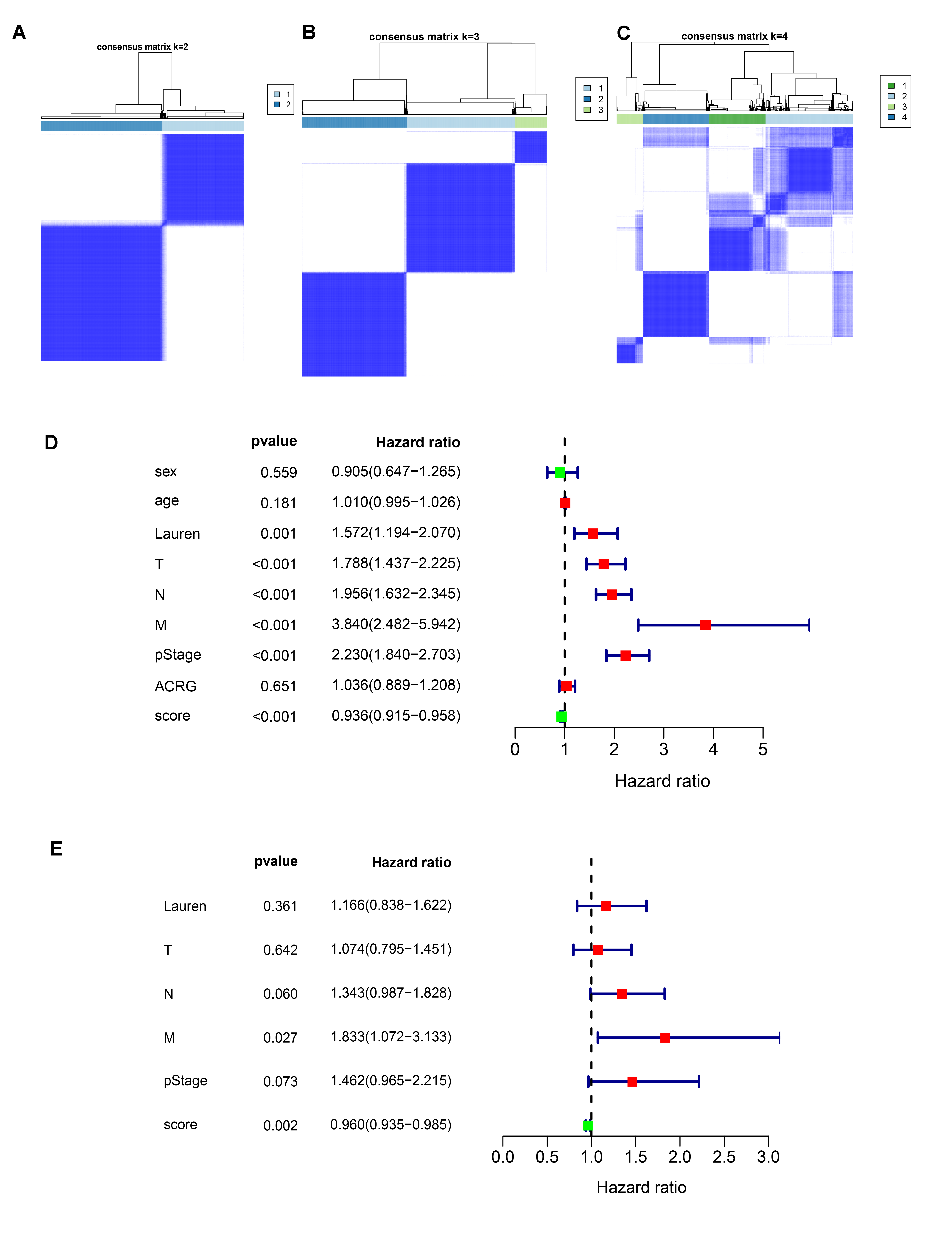

Supplement: Supplementary file 3 — Supplementary Figure 3 [file CAM4-12-879-s010.tif]

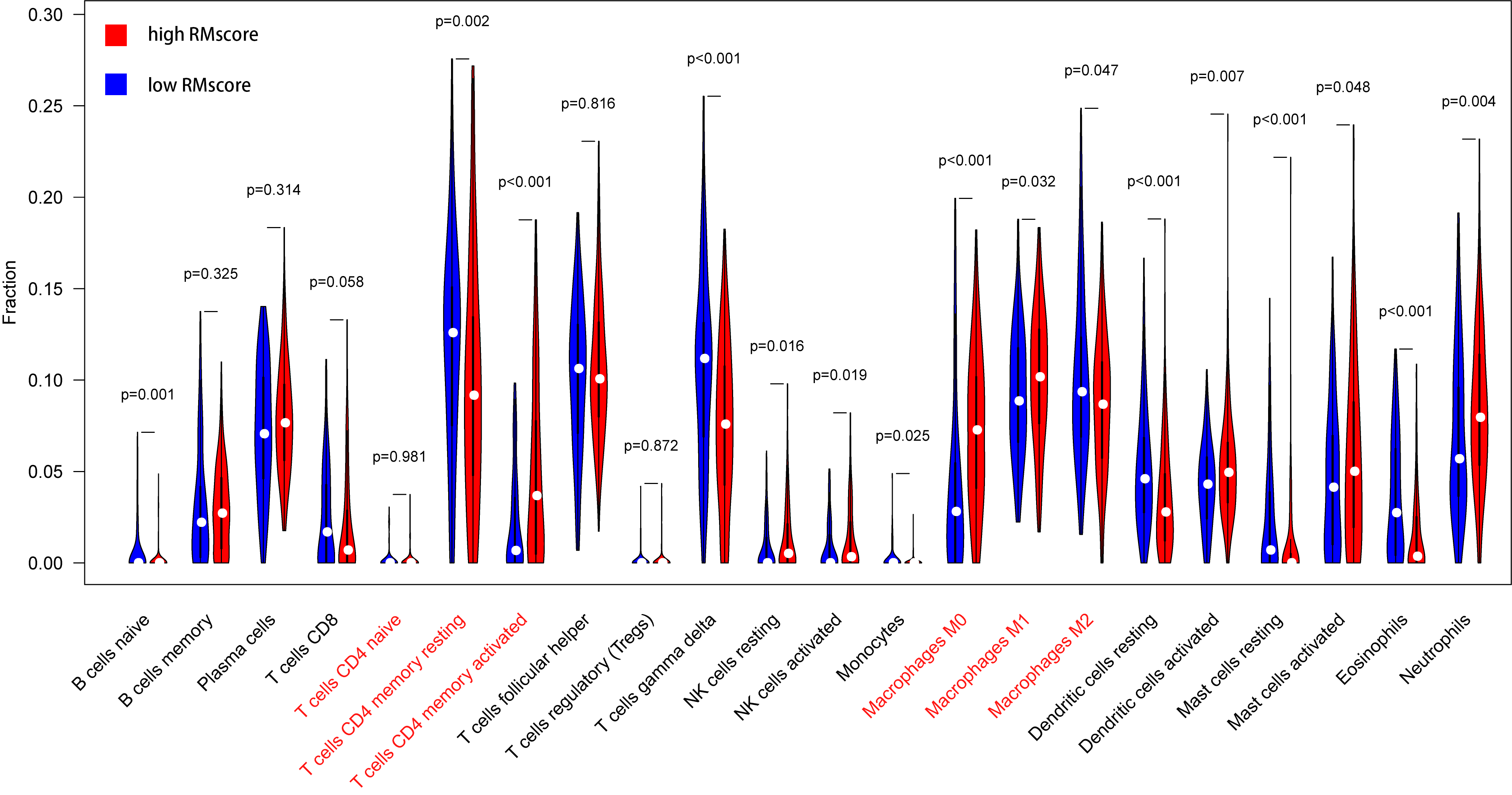

Supplement: Supplementary file 4 — Supplementary Figure 4 [file CAM4-12-879-s007.tif]
